# Chronotype is associated with eating behaviors, physical activity and overweight in school-aged children

**DOI:** 10.1186/s12937-023-00875-4

**Published:** 2023-10-06

**Authors:** Yong Yang, Si-Xuan Li, Yan Zhang, Fei Wang, Dan-Jie Jiang, Si-Jia Wang, Peng Cao, Qing-Hai Gong

**Affiliations:** 1 The First Affiliated Hospital of Ningbo University, Ningbo, Zhejiang, 315020 China; 2 Ningbo Municipal Center for Disease Control and Prevention, Ningbo, Zhejiang, 315010 China

**Keywords:** Chronotype, Eating behavior, Physical activity, Overweight, Children, China

## Abstract

**Background:**

A later chronotype has been found to be associated with unhealthy habits and diseases, such as an unhealthy diet and metabolic syndrome in adults. Little is known about the association between chronotype, eating habits, physical activity and obesity. Thus, this study aimed to explore the relationships between chronotype, eating behaviors, physical activity, and overweight in Chinese school-aged children.

**Methods:**

Data from this study was based on 952 schoolchildren (10–12 y) from six primary schools that participated in China. Anthropometric measurements of height and body weight were performed. Information about sleeping habits, dietary behaviors, and other lifestyle behaviors was gathered using a self-administered questionnaire. Multiple linear regression analysis or multivariable logistic regression model was performed to assess the associations between chronotype, eating behaviors, physical activity, and overweight.

**Results:**

Nearly 70% (69.9%) of the participants had a self-reported morning chronotype. Multiple linear regression analysis revealed chronotype score was positively associated with physical activities (all *P* values < 0.001) and sleep duration (all *P* values < 0.001) and negatively associated with BMI, meal time, eating jet lag and social jet lag (all *P* values < 0.001). Multivariable logistic regression analysis showed that compared to morning types, non-morning types individuals were more likely to be overweight (OR = 1.593, *P* value < 0.05), and had more frequent consumption of fast food (OR = 1.616, *P* value < 0.05), but less frequent consumption of milk (OR = 0.716, *P* value < 0.05), less time taking part in moderate (OR = 1.356, *P* value < 0.05) or muscle strengthening (OR = 1.393, 1.877, *P* value < 0.05) physical activity.

**Conclusions:**

This study indicates that early chronotype children are more active, have healthier dietary habits, get more sleep, have shorter social jet lag, and are less likely to be overweight than non-early chronotype children. Our findings suggest that later chronotype may be a potential indicator in the early detection of overweight, unhealthy eating, and physical inactivity behaviors.

**Plain english summary:**

Chronotype has been found to have an important impact on individual’s health. In the present study, we conducted a cross-sectional study to investigate the association between chronotype, eating behaviors, physical activity, and overweight in school-aged children. The findings showed that children with early chronotype is associated with more active, healthier dietary behaviors, longer sleep duration, short social jet lag, and a lower risk of overweight.

## Background

Chronotype is a measure of an individual’s circadian preferences that reflects the organization of the circadian system. There are three different types of chronotypes: Morning-types (M-types), Evening-types (E-types), and neither/Intermediate-types (N- types) [1]. Usually, chronotype is evaluated by self-assessment questionnaires. The most widely used and cited questionnaire is the Morningness–Eveningness Questionnaire (MEQ), which was developed by Horne and Ostberg in 1976 [2]. An evening chronotype or eveningness is associated with a circadian phase delay, which is hypothesized to be a good mark for circadian system dysfunction [3]. Biological rhythm is closely related to diet behaviors, physical activities and metabolism. Previous studies have shown that chronotype was associated with social jet lag (the misalignment between social and biological time, measured as the difference in sleep midpoint between work or school days and free days) [4, 5]. One research reported that adults with a later chronotype may have a greater social jet lag [6]. Another study reported that the timing of eating was also related to biological rhythm [7]. Recent research has reported that the evening chronotype was associated with unhealthy eating habits, such as nighttime eating behavior [8], binge-eating behavior [9], less consumption of fish and fruit, and greater consumption of soft drinks [10]. Similarly, survey-based studies indicated that the evening chronotype was associated with significantly lower levels of physical activity, but more sedentary behaviors, compared to the morning type [11, 12]. Additionally, a later self-reported chronotype was related to being overweight [13–15]. Up to now, few studies have examined the relationship between chronotype and overweight (or body mass index) in children [16–18].

In view of the fact that little is known about the associations between chronotype and social jetlag, eating behaviors, physical activity, and overweight among children, the objective of the present study was to examine these relationships among Chinese children. We hypothesized that, compared with non-morning types, children with morning chronotype would show healthier eating behaviors, more frequency of physical activity, and a lower risk of being overweight, even after adjustment for potential confounding factors.

## Methods

### Design and study population

This was a cross-sectional study, which was conducted by the Ningbo Municipal Center for Disease Control and Prevention (Ningbo CDC) to examine the relationships between health-related behaviors and chronic diseases in school children. This study was carried out in Ningbo City, Zhejiang Province, Mainland of China. A questionnaire survey and a physical examination were conducted by trained research staff during the same week in this study. Participants were recruited by using a non-random, purposive sampling from six primary schools in Ningbo City from April1. to April 26, 2021. To be eligible for this study, participants had to be ≧ 10 years of age, stay at the target school, and be able to independently complete the self-administered questionnaire survey. The only exclusion criteria were children’s refusal or inability to provide the information required for the development of the study. To estimate the sample size, we targeted sample size to be 600, which is the recommended minimum sample size guidelines for observational studies using logistic regression (based on the Events Per Variable criterion (EPV) of 30) [19]. Finally, a total of 1,430 schoolchildren (4th and 5th grades) aged 10–12 years were recruited for this study. All study participants and their parents or legal guardians provided written informed consent.

### Independent variables

#### Chronotype

Chronotype was assessed using the Morning and Evening Questionnaire (MEQ). The MEQ is the most widely used instrument for the identification of circadian preference [20]. The total score of all 19 items scales ranges from 16 to 86 points. According to the score, chronotype can be divided into three types: Evening type (16–41 points), Intermediate type (42–58 points), and Morning type (59–86 points). People with a higher score indicate a tendency to do morning activities. The Chinese version scale has been consolidated as a reliable and valid instrument [20, 21]. Owitng to the small number (n = 8) of participants in the ‘Evening type’ category, we combined the ‘Evening type’ and ‘Intermediate type’ categories into the category of ‘Non-Morning type’.

### Dependent variable

#### Sleeping behaviors

Sleep duration was assessed by a self-reported questionnaire including questions ‘During weekdays/weekends: what time do you usually go to bed?’, ‘During weekdays/ weekends: what time do you usually wake up?’. Average sleep duration was calculated as follows: [5×weekday sleep duration+2×weekday sleep duration]/7 [22]. Weekdays or weekends sleep duration was calculated by the absolute difference between self-reported bedtimes and wake-up times [23]. Social jet lag is primarily the result of a discrepancy between the sleep-wake cycle on weekdays and weekends, which was usually calculated based on the absolute difference between the mid-sleep time on weekends and weekdays [24]. We calculated mid-sleep time on weekend and weekdays as follow: bedtime + the middle of sleep length, for weekends and weekdays.

#### Eating behaviors

Timing of breakfast, lunch, and dinner on weekdays and weekends was estimated by a self-reported questionnaire including the questions’ During weekdays/weekends: what time do you usually eat breakfast/ lunch/ dinner?’ The eating midpoint was defined as the middle time point between the first and the last meal. The eating jet lag concept was proposed by a Spanish researcher, which was used as a marker of variability in meal timing, and was calculated based on the absolute difference between eating midpoints on weekends and weekdays [22]. To calculate the eating midpoint, first, we estimated the eating midpoint for weekdays and weekends as follows: Eating midpoint (local time) = ([Timing of the last meal- Timing of the first meal]/2) + Timing of the first meal. Food intake was assessed by the following questions: ⑴ During the past 7 days, did you eat or drink breakfast every day / sugary drinks more than one day / fast food more than one day? (Yes, No); ⑵ During the past 7 days, did you eat or drink green vegetables/ fruit/ milk more than one time per day? (Yes, No).

#### Physical activity

Physical activity was assessed by the questions ‘During the past 7 days, how many days did you participate in at least 60 min per day of light/moderate/strenuous physical activity?’ (1–7 days). Light physical activities involve minimal effort and no sweating, such as easy walking, walking or climbing stairs, or doing some cleaning. Details of questions relating to moderate and strenuous physical activities were described in our previous study [25]. A crude and a multivariate logistic regression analysis were performed with dependent variable low (< 2 days/ week) vs. normal physical activity (≥ 2 days/week) [26].

#### Anthropometric parameters

All participants’ heights and body weights were measured by trained research staff. Heights were measured to the nearest 0.1 cm with a free-standing stadiometer mounted on a rigid tripod (GMCS-I, Xindong Huateng Sports Equipment Co., Ltd., Beijing, China). Body weight was measured to the nearest 0.1 kg on a numerical scale (RGT-140, Weighing Apparatus Co., Ltd., Changzhou, China). Some details of the measurement have been described in a previous report [27]. BMI was computed using the ratio of weight/height^2^ (kg/m^2^). Overweight (including obesity) was defined according to age- and sex-specific cut-off points specified by the International Obesity Task Force (IOTF) [28].

### Confounding variables

Potential confounding covariates were drawn from previous studies and collected as a part of a self-administrated questionnaire, which covers age, gender (male, female), parental marital status, and parental education level on college [25].

### Statistical analysis

For the purpose of this study, data were divided into two groups according to their MEQ scores (Non- Morningness or Morningness). Continuous variables were presented as means (standard deviations), and categorical variables were analyzed using frequency and percentages. Differences in continuous variables were examined using Pearson’s *t*-tests, whereas categorical variables were analyzed using a chi-square test. Multiple linear regression analysis was used to evaluate the association between the chronotype score and social jet lag, mealtimes, physical activity, and body mass index after adjusting for potentially confounding factors (age, sex, parental marital status, and parental education level on college). Multiple regression models were checked for normality, multicollinearity and homoscedasticity. In addition, multivariable logistic regression analysis was performed to estimate the odds ratios (ORs) and 95% confidence intervals (CIs) of overweight, health-related eating behaviors and physical activities by chronotype adjusted for some potential confounding factors. The potential confounding variables in multivariable logistic regression models were selected based on health knowledge of variables that have been examined in previous research on cognitive style, and these variables showed significant associated with chronotype. In the logistic regression analysis, model 1 was unadjusted; and model 2 adjusted for potential confounding factors, such as age, gender, parental marital status and parental education level on college; and model 3 additionally adjusted for physical activity or eating behaviors, average sleep duration and social jet lag. Deviance (-2LL) were used for checking model fitness. Before conducting the regression models, we checked multicollinearity among all independent variables. All analyses were conducted with the PASW STATISTICS, version 26.0 (SPSS Inc., Chicago, IL, USA), and a two-tailed *P* value < 0.05 was considered statistically significant.

## Results

### Participants’ characteristics

A total of 952 (66.57%) students provided complete information in the present study. Information of the study sample characteristics according to the chronotype is shown in Table 1. The mean age of the sample was 10.20 ± 0.40 years and 48.4% were girls. Nearly 70% (69.9%) of the respondents had a self-reported morning chronotype. Morning types were more significantly associated with younger age, lower BMI, eating earlier, less eating jet lag, more frequencies of milk intake, less sugary drinks and fast food intake, more physical activities, longer sleep duration, and less social jet lag than non-morning chronotype (all *P* values < 0.05).

**Table 1:** Participants characteristics according to chronotypes (morningness-eveningness questionnaire score) (n = 952)

		Chronotype			*P* value
Characteristics	Total (n = 952)		Morning (n = 666)	Non-Morning (n = 286)	
Age (years, means ± SD)	10.20 ± 0.40		10.21 ± 0.41	10.18 ± 0.38	**0.034**
Sex (girls, %)	48.42		50.30	44.06	0.077
Anthropometric variables:					
Body mass index (kg/m^2^, means ± SD) Overweight (%)	17.43 ± 3.31 17.75%		17.21 ± 3.22 15.17	17.94 ± 3.46 23.78	**0.002** **0.001**
Parental marital status (married, %)	90.23		90.54	89.51	0.624
Parental education level on college (%)					0.678
Both had a college degree	39.39		43.84	45.10	
Only one of them had a college degree	16.39		15.92	17.48	
None of them had a college degree	44.22		40.24	37.41	
Meal time (h:min, means ± SD)					
Breakfast time weekdays	7:01 ± 17		6:59 ± 16	7: 03 ± 19	**0.012**
Breakfast time weekends	8:07 ± 39		8: 03 ± 39	8:18 ± 39	**< 0.0001**
Lunch time weekday	11:35 ± 22		11: 34 ± 21	11:39 ± 24	**0.003**
Lunch time weekends	11:40 ± 30		11: 44 ± 36	11:49 ± 34	**< 0.0001**
Dinner time weekday	17:57 ± 31		17:56 ± 30	17:59 ± 33	0.097
Dinner time weekends	17:56 ± 33		17:54 ± 33	18:00 ± 34	**0.013**
Weekdays Eating midpoint	13:02 ± 35		12:58 ± 34	13:10 ± 38	**< 0.0001**
Weekends Eating midpoint	7:43 ± 44		13:37 ± 45	13:56 ± 45	**< 0.0001**
Eating jet lag (h, means ± SD)	0.73 ± 0.47		0.69 ± 0.47	0.82 ± 0.45	**< 0.0001**
Breakfast skipping ( ≦ 6 days/ week, %)	6.51		6.01	7.69	0.334
Eating behaviors (%)					
Green vegetables intake( ≧ 1 times /week)	72.90		74.17	69.93	0.177
Fruit intake ( ≧ 1 times/day)	74.68		76.43	70.63	0.059
Milk intake ( ≧ 1 times/day)	57.98		60.36	52.45	**0.023**
Sugary drinks ( ≧ 1 days/week)	44.43		42.19	49.65	**0.034**
Fast food ( ≧ 1 days/week)	30.57		27.18	38.46	**0.001**
Physical activity (days/week, means ± SD)					
Light physical activity 60 min a day	2.96 ± 2.17		3.12 ± 2.22	2.61 ± 2.04	**0.001**
Moderate physical activity 60 min a day	2.28 ± 1.97		2.42 ± 2.04	1.96 ± 1.78	**0.001**
Muscle strengthening activity 60 min a day	2.66 ± 2.31		2.84 ± 2.38	2.22 ± 2.06	**0.001**
Circadian parameters (h:min, means ± SD)					
Bedtime weekdays	21:20 ± 33		21:16 ± 33	21:30 ± 33	**< 0.0001**
Wake time weekdays	6:41 ± 18		6:40 ± 18	6:44 ± 18	**< 0.0001**
Bedtime weekends	21:40 ± 38		21:34 ± 38	21:53 ± 34	**< 0.0001**
Wake time weekends	7:43 ± 44		7:37 ± 41	7:58 ± 46	**< 0.0001**
Average sleep duration (h/night, means ± SD)	9.26 ± 0.53		9.32 ± 0.38	9.13 ± 0.46	**< 0.0001**
Sleep duration in weekdays (h/night, means ± SD)	9.35 ± 0.55		9.40 ± 0.54	9.24 ± 0.56	**< 0.0001**
Sleep duration in weekends (h/night, means ± SD)	9.03 ± 0.63		9.10 ± 0.62	8.86 ± 0.60	**< 0.0001**
Social jetlag (h, means ± SD)	0.40 ± 0.35		0.38 ± 0.35	0.46 ± 0.34	**< 0.0001**

Bold: *P* values<0.05

### The association between chronotype and eating behaviors, physical activity and overweight

Table 2 shows the multiple linear regression analyses examining associations of chronotype score with Social Jet Lag, meal timing, physical activity, and BMI. After adjusting for age, sex, parental marital status, and parental education at the college level, the chronotype score was positively associated with frequency of light physical activity(β = 0.041; 95%CI: 0.022, 0.060, *P* < 0.001), moderate physical activity (β = 0.040; 95%CI: 0.023, 0.057, *P* < 0.001), muscle strengthening activity (β = 0.049; 95%CI: 0.029, 0.068, *P* < 0.001), average sleep duration (β = 0.014; 95%CI: 0.009, 0.018, *P* < 0.001), sleep duration in weekdays (β = 0.013; 95%CI: 0.008, 0.017, *P* < 0.001), and sleep duration in weekends(β = 0.017; 95%CI: 0.012, 0.023, *P* < 0.001), but was negatively associated with BMI (β= -0.056; 95%CI: -0.085, -0.028, *P* < 0.001), breakfast time weekdays (β= -0.004; 95%CI: -0.007, -0.002, *P* < 0.001), breakfast time weekends (β= -0.026; 95%CI: -0.032, -0.021, *P* < 0.001), lunch time weekdays (β= -0.007; 95%CI: -0.010, -0.004, *P* < 0.001), lunch time weekends (β= -0.013; 95%CI: -0.017, -0.009, *P* < 0.001), dinner time weekdays (β= -0.009; 95%CI: -0.013, -0.004, *P* < 0.001), dinner time weekends (β= -0.009; 95%CI: -0.014, -0.004, *P* < 0.001), eating jet lag(β= -0.012; 95%CI: -0.016, -0.008, *P* < 0.001) and social jet lag (β= -0.007; 95%CI: -0.010, -0.004, *P* < 0.001) .

**Table 2:** Associations of chronotype score (MEQ^a^) with social jet lag, sleep duration, meal timing, physical activity and body mass index (BMI)^b^

Characteristics	β^c^	95% CI^d^	*P* value^e^
Body mass index(kg/m^2^)	-0.056	-0.085, -0.028	**< 0.0001**
Meal timing			
Breakfast time weekdays (h:min)	-0.004	-0.007, -0.002	**0.001**
Breakfast time weekends (h:min)	-0.026	-0.032, -0.021	**< 0.0001**
Lunch time weekdays (h:min)	-0.007	-0.010, -0.004	**< 0.0001**
Lunch time weekends (h:min)	-0.013	-0.017, -0.009	**< 0.0001**
Dinner time weekdays (h:min)	-0.009	-0.013, -0.004	**< 0.0001**
Dinner time weekends (h:min)	-0.009	-0.014, -0.004	**< 0.0001**
Weekdays Eating midpoint (h:min)	-0.016	-0.021, -0.011	**< 0.0001**
Weekends Eating midpoint (h:min)	-0.027	-0.033, -0.021	**< 0.0001**
Eating jet lag (h)	-0.012	-0.016, -0.008	**< 0.0001**
Physical activity			
Light physical activity 60 min a day (days/week)	0.041	0.022, 0.060	**< 0.0001**
Moderate physical activity 60 min a day (days/week)	0.040	0.023, 0.057	**< 0.0001**
Muscle strengthening activity 60 min a day (days/week)	0.049	0.029, 0.068	**< 0.0001**
Average sleep duration (h/night)	0.014	0.009, 0.018	**< 0.0001**
Sleep duration in weekdays (h/night)	0.013	0.008, 0.017	**< 0.0001**
Sleep duration in weekends (h/night)	0.017	0.012, 0.023	**< 0.0001**
Social jet lag (h)	-0.007	-0.010, -0.004	**< 0.0001**

^a^ MEQ, Morning-Evening Questionnaire

Data are presented as unstandardized beta coefficients (standard error)

Chronotype score was expressed as a continuous variable ranging from 16 to 86, higher score indicates a preference for morningness. CI, confidence interval

^b^ Models adjust for children age, sex, parental marital status and parental education level on college

^c^ β: standardized regression

^d^ c: confidence interval

^e^*P* value from the multiple linear regression model

Bold: P values<0.05

Table 3 presents the results of logistic regression analyses regarding the associations between chronotype and overweight. After adjusting for age, sex, parental marital status and parental education level on college, physical activity, sleep duration, social jet lag and eating jet lag, non-morning types were more likely to be overweight (OR = 1.530; 95%CI: 1.111, 2.285, *P* < 0.05).

**Table 3:** Logistic regression model predicting overweight from chronotype (MEQ score ^a^)

	Chronotype				
	Morning type Non-Morning type				
	Ref. ^b^	-2LL^c^	OR ^d^	95%CI ^e^	*P* –value ^f^
Total number (n)	666			286	
Overweight (n)	101			68	
Model 1	1.00	880.585	1.745	1.236, 2.464	**0.002**
Model 2	1.00	860.539	1.697	1.196, 2.408	**0.003**
Model 3	1.00	854.039	1.593	1.111, 2.285	**0.011**

^a^ MEQ, Morning-Evening Questionnaire

^b^ Ref., Reference; ^c^ -2LL, -2 log likelihood; ^d^ OR, odds ratio; ^e^ CI, confidence interval; *P* –value ^f^, *p* value from the logistic regression model

Model 1: unadjusted

Model 2: adjusted for age, sex, parental marital status and parental education level on college

Model 3: model 2 + physical activity, average sleep duration, social Jet Lag and eating Jet Lag

Bold: *P* values<0.05

Table 4 shows the association between eating behaviors, physical activities and chronotype were analyzed using multivariable logistic regression models. After adjusting for some potential confounders, the non-morning types were observed to be more likely to have more frequent consumption of fast food (OR = 1.616; 95%CI: 1.191, 2.193, *P* < 0.05), but less frequent consumption of milk (OR = 0.716; 95%CI: 0.534, 0.959, *P* < 0.05), and less time taking part in moderate (OR = 1.356; 95%CI: 1.015, 1.813, *P* < 0.05) or muscle strengthening (OR = 1.393; 95%CI: 1.034, 1.877, *P* < 0.05) physical activity than morning types.

**Table 4:** Logistic regression model predicting eating behaviors and physical activities from chronotype (MEQ score ^a^)

	Chronotype				
	Morning type Non-Morning type				
	Ref. ^b^	-2LL^c^	OR ^d^	95%CI ^e^	*P* –value ^f^
Total number (n)	666			286	
Breakfast skipping (n) ( ≦ 6 days/ week)	40			22	
Model 1	1.00	457.661	1.304	0.760, 2.237	0.335
Model 2	1.00	435.572	1.362	0.786, 2.361	0.270
Model 3	1.00	418.436	1.065	0.602, 1.884	0.828
Vegetables consumption (n) ( ≧ 1 times /week)	492			200	
Model 1	1.00	1110.627	0.810	0.596, 1.100	0.177
Model 2	1.00	1106.809	0.818	0.601, 1.113	0.201
Model 3	1.00	1096.064	0.790	0.573, 1.088	0.148
Fruit consumption ( ≧ 1 times /week) (n)	509			202	
Model 1	1.00	1073.735	0.742	0.543, 1.102	0.060
Model 2	1.00	1071.053	0.747	0.546, 1.021	0.067
Model 3	1.00	1063.313	0.796	0.576, 1.099	0.165
Fast food consumption ( ≧ 1 times /week) (n)	181			110	
Model 1	1.00	1160.349	1.675	1.249, 2.245	**0.001**
Model 2	1.00	1154.815	1.678	1.249, 2.253	**0.001**
Model 3	1.00	1145.429	1.616	1.191, 2.193	**0.002**
Milk intake ( ≧ 1 times/day) (n)	402			150	
Model 1	1.00	1290.264	0.724	0.548, 0.958	**0.024**
Model 2	1.00	1277.879	0.708	0.534, 0.939	**0.016**
Model 3	1.00	1268.549	0.716	0.534, 0.959	**0.025**
Sugary drinks consumption ( ≧ 1 times /week) (n)	281			142	
Model 1	1.00	1303.431	1.351	1.023, 1.784	**0.034**
Model 2	1.00	1298.057	1.346	1.018, 1.781	**0.037**
Model 3	1.00	1294.861	1.314	0.985, 1.753	0.064
Physical activity					
Light physical activity 60 min a day (< 2 days/week) (n)	176			100	
Model 1	1.00	1139.406	1.497	1.111, 2.016	**0.008**
Model 2	1.00	1120.926	1.485	1.098, 2.008	**0.010**
Model 3*	1.00	1105.710	1.344	0.983, 1.839	0.064
Moderate physical activity 60 min a day (< 2 days/week) (n)	271			145	
Model 1	1.00	1296.474	1.499	1.134, 1.981	**0.004**
Model 2	1.00	1292.795	1.508	1.140, 1.996	**0.004**
Model 3*	1.00	1278.460	1.356	1.015, 1.813	**0.040**
Muscle strengthening activity 60 min a day (< 2 days/week) (n)	260			139	
Model 1	1.00	1287.261	1.477	1.117, 1.952	**0.006**
Model 2	1.00	1242.800	1.492	1.120, 1.988	**0.006**
Model 3*	1.00	1231.910	1.393	1.034, 1.877	**0.029**

^a^ MEQ, Morning-Evening Questionnaire

^b^ Ref., Reference; ^c^ -2LL, -2 log likelihood; ^d^ OR, odds ratio; ^e^ CI, confidence interval; *P* –value ^f^, *p* value from the logistic regression model

Model 1: unadjusted

Model 2: adjusted for age, sex, parental marital status and parental education level on college

Model 3: model 2 + physical activity, average sleep duration, social Jet Lag and eating Jet Lag

Model 3*: model 2 + milk intake, sugary drinks, fast food consumption, eating Jet Lag, average sleep duration and social Jet Lag

Bold: *P* values<0.05

## Discussion

To our knowledge, this is the first study to assess the potential associations between chronotype and objective BMI, meal timing, physical activity, sleep duration, social jet lag, and eating behaviors in a relatively large sample of Chinese school children. The results from this cross-sectional study showed that non-morning chronotypes had a significantly higher risk of being overweight, poorer sleep, longer social jet lag and eating jet lag, some unhealthy dietary behaviors, and a lower frequency of physical activities compared to morning chronotypes.

Chronotype is defined as a trait determining the individual’s preferences in behavioral and biological rhythms, which play an integral role in diverse aspects of an individual’s life [29]. Some literature has reported that children who had delayed sleep timing or short sleep duration were associated with a higher BMI and a greater risk of obesity [30–32]. There is limited research examining the association between chronotype and BMI or overweight in children [14, 17, 33–36]. In addition, our results show that a later chronotype was associated with later meal timing, more unhealthy behaviors and eating patterns. These factors may play a role in mediating the potential effect of chronotype on BMI. Our logistic regression analyses appear to follow the results of the majority of previous studies [33, 34]. However, similar to other studies, we did not have a direct measurement of children’s energy expenditure or energy balance, making it difficult to exclude potential confounding factors in the relationship between chronotype and overweight.

Evidence has shown that people who tend to have a morning-type chronotype appear to have healthy eating behaviors, such as being less likely to skip breakfast [37], eating early in the day [38], less consumption of high-fat snacks or soda drinks [39], higher diet quality [40], and shorter eating jet lag [22]. Our present study is in agreement with four previous reports [22, 24–26, 37, 38]. Interestingly, we found that, compared with morningness, children with a non-morning chronotype tend to more consumption of fast food, but lower consumption of milk. However, the underlying mechanism of this relationship is not yet clear. It is possible that non-morning chronotypes are driven by misalignment of lifestyle behaviors with the circadian misalignment, which could cause increased responsiveness to rewarding food properties or changes in gut signaling promoting appetite [41]. However, it is possible that our sample size might not have been sufficient to detect the hypothesized significant relationships between chronotype and the consumption of sugary drinks, vegetables, and fruit. Studies with a larger sample size in the future are required.

Noteworthy, the results of our study showed that children with a morning type preferred earlier sleep/wake timing, and less variability in sleep timing during weekends versus weekdays. They may affect eating timing or eating schedules. This may explain why some of the eating jet lag in children with a morning type was lower than others. Given the results of the above-mentioned research, our study hypothesizes that delaying sleep timing on weekdays and weekends would be in line with their circadian timing of eating. Previous studies have suggested that people with a more evening-type chronotype tend to breakfast skipping [37, 38] and consume a higher total energy intake [40, 42], this could at least partly explain the later chronotype was associated with overweight among schoolchildren in our study. Two previous studies investigated associations between chronotype or sleep timing and physical activity and found that later chronotype was associated with lower levels of physical activity among children and adolescents [43, 44]. The results of this study supported previous research that children with a morning chronotype tend to have a higher frequency of moderate or muscle strengthening physical activity. It is possible to hypothesize that evening types have more difficulties in finding a suitable time [45] and more sleep problems, and daytime tiredness [46] than those with morning types.

The present study’ results suggest that future prevention of obesity, unhealthy of eating, and leisure physical activities, can be achieved through multipath interventions such as adherence to a fixed advanced sleep schedule. Our present study also has several strengths that should be noted. First, chronotype was assessed using a validated method [21] and both multiple linear regression and multivariable logistic regression analyses were adjusted for several potential confounding factors. Furthermore, this study included a relatively large sample size of participants, and objectively determined body weight and height. However, our study also has some limitations. First, as a result of the cross-sectional nature, it was not possible to identify the temporal relationships. Second, measures of chronotype, meal timing, dietary behaviors, physical activity, and sleep timing were derived from a self-report questionnaire, which could introduce measurement errors. Finally, we did not measure other important study variables, such as total daily energy intake and family economic status, which may be related to eating behaviors, physical activity, and BMI.

## Conclusion

In conclusion, this study provides a novel insight into the association of chronotype with physical activity, eating behaviors, and overweight in Chinese school-aged children, showing that early chronotype is associated with more active, healthier dietary behaviors, longer sleep duration, short social jet lag, and a lower risk of overweight. Our findings indicate that it is important to assess the sleep schedule in children, as it may be a potential indicator in the early detection of overweight, unhealthy eating, and physical activity behaviors. Further research on the mechanisms and large-scale prospective studies are needed to verify our results.

## Data Availability

Additional data can on request be made available.
